# Mechanistic Insights into Antioxidant Interventions Targeting Obesity-Induced Oxidative Stress in the Pathogenesis and Complications of Type 2 Diabetes Mellitus

**DOI:** 10.3390/cimb47121063

**Published:** 2025-12-18

**Authors:** Fani-Niki Varra, Panagiotis Theodosis-Nobelos, Viktoria-Konstantina Varra, Michail Varras

**Affiliations:** 1 Department of Pharmacy, School of Health Sciences, Frederick University, Nicosia 1036, Cyprus; fannyvarra@gmail.com (F.-N.V.); hsc.np@frederick.ac.cy (P.T.-N.); 2 Medical School, Democritus University of Thrace, 68100 Alexandroupolis, Greece; 3 Department of Pharmacy, School of Health Sciences, National and Kapodistrian University of Athens, Panepistimiopolis of Zographou, 15771 Athens, Greece; nadv.ars2016@gmail.com; 4 Division of Aesthetics and Cosmetic Science, Department of Biomedical Sciences, School of Health and Welfare Sciences, University of West Attica, 12243 Athens, Greece; 5 Fourth Department of Obstetrics and Gynecology, ‘Elena Venizelou’ General and Maternity Hospital, 11521 Athens, Greece

**Keywords:** obesity, oxidative stress, antioxidants, natural antioxidants, vitamins, catechins, flavonoids, adipocytes, cardiovascular disease, diabetes

## Abstract

Diabetes mellitus (DM) is a complex, heterogeneous, hyperglycemic chronic metabolic disorder. Type 2 diabetes mellitus (T2DM) is characterized by progressive loss of insulin secretion from pancreatic islet β-cells due to IR (insulin resistance), which is a feature of metabolic syndrome (MetS). Chronic hyperglycemia in patients with T2DM in synergy with other metabolic abnormalities causes complications such as diabetic ketoacidosis, osmotic diuresis and hyperglycemic diabetic coma, as well as chronic microvascular and macrovascular complications such as atherosclerotic cardiovascular disease (ASCVD), peripheral artery disease (PAD) and cerebrovascular events, which implicate the formation of reactive species and the promotion of inflammatory pathways. In these events, natural or synthetic antioxidants and minerals seem to have ameliorative effects and may serve as beneficial co-treatment options. In view of these terms, the aim of this study is to investigate the underlying mechanisms of T2DM, its clinical presentation, and its complications. Additionally, the association of the pathogenesis of T2DM and the occurrence of its complications with obesity, chronic inflammation, oxidative stress (OS), insulin resistance (IR), hepatic steatosis, and dyslipidemia is examined, whilst molecular pathways, such as NF-κB and JAK/STAT, are also summarized, under the scope of the effects of several antioxidant compounds and minerals on their progression. The interrelation of T2DM with these conditions, as well as the effects of antioxidant supplementation, seems to be bidirectional, and it is recommended that obese patients be screened for T2DM and adopt lifestyle changes, including exercise, diet modification, and weight loss, in addition to potentially taking multifunctional supplements that offer antioxidant and anti-inflammatory potential. However, many aspects of the protective mechanisms of such antioxidants remain to be elucidated, with more drawbacks in their pharmacokinetic behavior, such as their poor absorption and solubility, waiting to be resolved.

## 1. Introduction

The term *diabetes* originates from the Greek verb diabaino, meaning “to pass through” [1]. The ancient physician Aretaeus of Cappadocia used the term *diabetes* to describe a condition characterized by excessive urination, or polyuria [1], with a distinction being made later between *diabetes mellitus* and *diabetes insipidus*, with the latter referring to a condition in which the urine is tasteless and lacks any distinctive quality [2]. Diabetes mellitus is a heterogeneous metabolic disorder characterized by chronic hyperglycemia resulting from impaired insulin secretion, defective insulin action, or a combination of both [3]. The most common classifications (types) include T1DM, T2DM, and gestational diabetes [4].

T2DM is among the most significant non-communicable chronic diseases, exhibiting high rates of morbidity and mortality [5,6]. It accounts for approximately 96% of all diabetes cases and is characterized by (a) non-autoimmune, progressive loss of adequate insulin secretion by pancreatic β-cells within the islets of Langerhans, (b) IR, and (c) MetS [7,8]. Furthermore, its strong association with obesity and the involvement of OS in both the onset and complications of T2DM are documented, with many aspects of their interrelated pathophysiology needing elucidation. Numerous inflammatory and oxidative mechanisms contribute to this relationship [9,10]. In light of the above, the present study aims to elucidate the pathways implicated in the development and progression of T2DM and its complications, emphasizing their interrelation with obesity and OS-related signaling. Furthermore, it seeks to analyze the impact of antioxidant vitamins, natural compounds, and minerals on these processes, drawing upon data from the international literature, mainly from the last decade, and comparing findings from *in cellulo*, in vivo, and clinical studies. The analysis of these antioxidants seeks to offer not only information for their potential usage as supplemental compounds for the conditions caused by T2DM, but also to shed light on the molecular mechanisms that are related to their progression and resolution.

## 2. Pathophysiology of Obesity-Induced IR and T2DM

**Figure 1:**
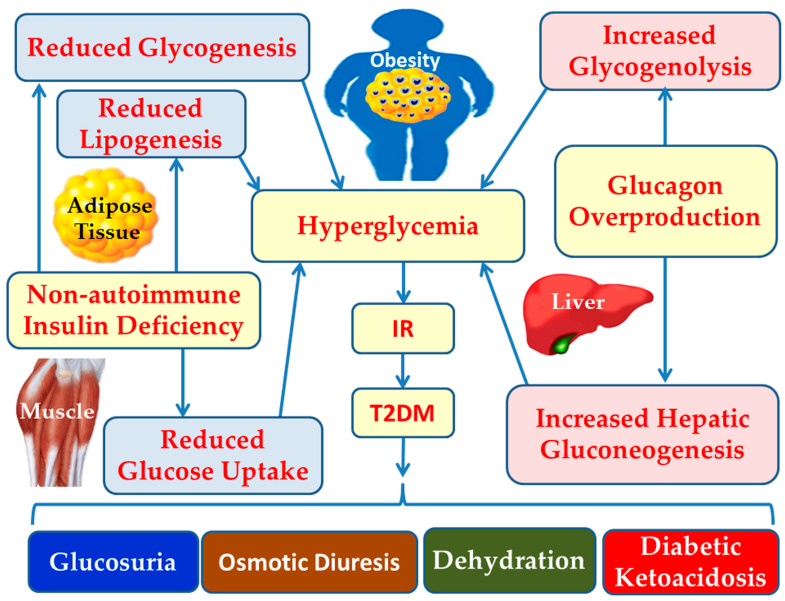
Illustration of the pathophysiologic mechanisms of obesity-induced IR and T2DM development: Obesity can cause non-autoimmune insulin deficiency through chronic low-grade inflammation, adipose tissue hypoxia, and OS, which lead to hyperglycemia, disruption of insulin signaling, IR, pancreatic β-cell dysfunction and T2DM development. At the same time, obesity can lead to overproduction of glucagon, which causes hyperglycemia and contributes to IR and T2DM development. Hyperglycemia in T2DM leads to glycosuria, which causes osmotic diuresis and dehydration. Also, T2DM can lead to the breakdown of fat for energy production, generating ketone bodies and potentially causing diabetic ketoacidosis.

Hyperglycemia in T2DM results from reduced glucose utilization due to non-autoimmune insulin deficiency and the excessive production of glucagon [11]. Insulinopenia leads to decreased glucose uptake by muscle and adipose tissues, reduction in glycogen synthesis, and diminished lipogenesis [6]. Excessive glucagon secretion, which becomes the dominant hormone in carbohydrate metabolism, promotes increased glycogenolysis and gluconeogenesis in the liver [12]. Among these mechanisms, elevated hepatic gluconeogenesis (mediated by glucagon activity) contributes more significantly to hyperglycemia than impaired glucose utilization [13]. The ensuing consequences of hyperglycemia include glucosuria, osmotic diuresis, dehydration, and diabetic ketoacidosis [14] (Figure 1).

### 2.1. Insulin Signaling Pathways, IR, and the Pathogenesis of T2DM

Under normal physiological conditions, insulin signaling begins with the phosphorylation of its cytoplasmic receptor and proceeds through two parallel pathways: (a) PI3K/AKT pathway and (b) the MAPK pathway [15]. Activation of AKT by insulin induces the translocation of the glucose transporter GLUT4 from cytoplasmic vesicles to the plasma membrane, thereby promoting glucose uptake [6]. In addition, AKT activation by insulin phosphorylates the transcription factor FOXO1 in the nucleus and translocates it to the cytoplasm, resulting in its inactivation and the suppression of hepatic gluconeogenesis [6,16]. Furthermore, insulin-induced AKT activation phosphorylates and inactivates GSK3, leading to enhanced glycogen synthesis, while it also stimulates mTOR activity, promoting protein synthesis [6]. Under certain conditions, insulin can also activate the MAPK pathway, leading to increased secretion of endothelin-1 from vascular endothelial cells and subsequent vasoconstriction [15].

IR refers to the reduced ability of insulin target tissues to respond to insulin signaling, promoting compensatory hyperinsulinemia, which is closely associated with MetS and related disorders such as obesity, NAFLD, and hypertension [6]. In the early stages of IR, pancreatic β-cells become hyperfunctional in an attempt to increase insulin secretion and maintain normal plasma glucose levels [17]. As the condition progresses, β-cells can no longer secrete sufficient insulin for the high blood glucose concentrations, leading to prediabetes or overt T2DM [18].

In adipose tissue, IR induces enhanced lipolysis, while in the liver it promotes lipogenesis and gluconeogenesis, resulting in compensatory hyperinsulinemia to maintain normoglycemia [15]. In skeletal muscle, IR impairs GLUT4 translocation to the plasma membrane, thereby reducing glucose internalization [15]. Increased lipolysis of visceral fat releases free fatty acids (FFAs) into the liver via the portal circulation, leading to elevated secretion of vLDLs and triglycerides. The accumulation of triglycerides and FFAs in the liver causes hepatic lipotoxicity and contributes to the development of NAFLD [15]. Moreover, elevated plasma FFAs contribute to increased LDL levels and decreased HDL concentrations, thus generating an atherogenic dyslipidemia [19]. In parallel, IR elevates serum fibrinogen and PAI-1 levels, promoting a prothrombotic state in T2DM, thereby increasing the risk of thrombosis and CVDs [20].

### 2.2. Obesity, Chronic Low-Grade Inflammation, OS and the Pathogenesis of T2DM

Obesity is linked to adipose tissue chronic low level inflammation. In obesity there is increased secretion of chemotactic factors, anti-inflammatory and pro-inflammatory adipokines, as well as pro-inflammatory cytokines [15]. This inflammatory process involves a phenotypic shift in adipose tissue macrophages, from anti-inflammatory M2 macrophages, predominant in lean individuals, to pro-inflammatory M1 macrophages, which dominate in overweight and obese subjects [15]. In individuals with normal body weight, adipose tissue macrophages are mainly of the M2 subtype, producing anti-inflammatory adipokines, such as adiponectin and apelin, and anti-inflammatory cytokines including IL-4, IL-10 and IL-11 [15]. Excessive caloric intake and physical inactivity lead to adipocyte hypertrophy, promoting increased secretion of chemotactic factors such as MCP-1 into the circulation, which in turn stimulates the recruitment and activation of pro-inflammatory M1 macrophages [21]. M1 macrophages infiltrate adipose tissue, resulting in the production of (i) pro-inflammatory adipokines such as visfatin, resistin, leptin, and PAI-1; (ii) pro-inflammatory cytokines such as IL-6, TNF-α, and IL-1β; and (iii) iNOS. The chronic inflammation that ensues leads to reduced adiponectin levels [15]. These pro-inflammatory adipokines and cytokines impair insulin signaling in the liver, adipose tissue, and skeletal muscle, while activating multiple inflammatory signaling cascades, including NF-κB, JAK/STAT, and JNK pathways. This molecular dysregulation promotes the development of MetS and T2DM [6].

In the liver, adipose tissue, and skeletal muscles, chronic hyperinsulinemia, secondary to persistent hyperglycemia, further diminishes insulin signaling. The infiltration of adipose tissue by M1 macrophages enhances the secretion of pro-inflammatory cytokines, perpetuating chronic inflammation and contributing to OS [15]. Moreover, hyperglycemia increases mitochondrial production of nicotinamide adenine dinucleotide phosphate (NADPH), which enhances the generation of ROS. These ROS exceed the capacity of endogenous antioxidant systems, thereby promoting OS [22]. Elevated ROS levels exacerbate OS and downregulate AKT activity, resulting in sustained hyperinsulinemia and IR. The latter further diminishes insulin signaling in the liver, adipose tissue, and skeletal muscles [15]. Concurrently, both increased secretion of pro-inflammatory cytokines from adipose tissue and chronic hyperinsulinemia lead to pancreatic β-cell dysfunction, leading to T2DM [15].

OS is also implicated in the pathogenesis of T2DM complications, including renal dysfunction, diabetic retinopathy, and diabetic peripheral neuropathy [6]. In diabetic nephropathy, chronic hyperglycemia induces excessive ROS accumulation within the renal mesangium, triggering activation of mTOR [6], and sirtuin 1 [23], ultimately leading to mesangial cell apoptosis [6,24]. Similarly, in diabetic retinopathy, persistent hyperglycemia causes excessive accumulation of ROS and AGEs in ocular tissues [25], activating the polyol, hexosamine, and PKC pathways, which culminate in retinal cell apoptosis and disease progression [26]. In diabetic peripheral neuropathy, both hyperglycemia and elevated SFAs contribute to mitochondrial membrane depolarization and ROS accumulation in SCs, leading to activation of downstream effectors such as PKCε [27] and JNK [28], and ultimately resulting in mitochondrial dysfunction and Schwann cell apoptosis [29].

### 2.3. The NF-κB Signaling Pathway in the Pathogenesis of T2DM and Its Complications

Proinflammatory cytokines secreted by adipose tissue in obese individuals with T2DM phosphorylate and degrade the IKK complex [30], thereby releasing and activating NF-κB, which subsequently translocates into the cell nucleus to exert its transcriptional activity [6]. NF-κB regulates immune responses, stress reactions, apoptosis, and cellular differentiation [31]. Specifically, NF-κB inhibits the activation of IRS-1 by increasing the expression of iNOS and the production of NO [6]. Moreover, IRS-1 activation is suppressed through serine phosphorylation by IKKβ, a subunit of the IKK complex. Additionally, NF-κB inhibits IRS-1 activation through the stimulation of PKCε via the NLRP3 inflammasome [6]. Concurrently, NF-κB upregulates the transcription of IL-1β, IL-18, and TNF-α, creating a positive feedback loop that further amplifies its activity and promotes metabolic syndrome-related comorbidities such as diabetic nephropathy, foot ulcers and retinopathy [6]. In diabetic nephropathy, NF-κB activation and its stimulation of the NLRP3 inflammasome promote renal inflammation and enhance the deposition of fibronectin and collagen in the kidney [32], leading to thickening of the glomerular basement membrane, glomerulosclerosis, podocyte injury, and ultimately renal fibrosis [33]. In diabetic foot ulcers, NF-κB activation induces wound inflammation and increases caspase-3 activity, resulting in apoptosis of vascular endothelial cells and delayed wound healing [34]. During the early stages of diabetic retinopathy, NF-κB activation interacts with ROS to promote pro-apoptotic processes [35]. In the later stages of PDR, abnormal neovascularization occurs, accompanied by apoptosis of pericytes in the retinal layer due to elevated levels of the NLRP3 inflammasome and IL-1β driven by NF-κB activity [36].

### 2.4. The JAK/STAT Signaling Pathway in the Pathogenesis of T2DM and Its Complications

The JAK/STAT signaling pathway serves as a key mediator in the downstream signaling of various cytokines, hormones, and growth factors, and plays a crucial role in regulating cellular proliferation, differentiation, apoptosis, and inflammatory responses [6]. In T2DM, elevated levels of proinflammatory cytokines, such as IL-6 and IFN-γ, activate the JAK2/STAT3 pathway, leading to the upregulation of SOCS3, which inhibits the activation IRS-1 [37,38]. Activation of the JAK/STAT3 pathway by these proinflammatory cytokines has also been found to induce NF-κB activation [37]. Furthermore, JAK/STAT3 signaling is implicated in T2DM-related complications reported above. In diabetic nephropathy, increased JAK expression in glomerular podocytes activates the STAT3/NF-κB axis, resulting in persistent low-grade renal inflammation, which promotes fibrosis and progressive loss of renal function [39,40]. In diabetic foot ulcers, elevated proinflammatory cytokines, such as IL-6, enhance STAT3 signaling [41], which impairs immune cell activation, recruitment, and survival, ultimately leading to delayed wound healing [42].

## 3. Pathophysiology of T2DM Complications

Patients with diabetes mellitus frequently present with excessive thirst (polydipsia), extreme hunger (polyphagia), increased urination (polyuria), lack of energy and fatigue, bacterial and fungal infections of the skin and genital area, dry mouth, xerosis, nausea, vomiting, abdominal pain, headache, facial flushing, dehydration, and delayed wound healing [43]. Some individuals may also report numbness or blurred vision [43]. Uncontrolled diabetes can lead to both acute and chronic complications. The former includes hypoglycemia, diabetic ketoacidosis, hyperglycemic hyperosmolar state, and hyperglycemic diabetic coma [43].

The latter include neuropathy and diabetic retinopathy, with their macrovascular complications including ASCVD, PAD, and cerebrovascular events [3,6,43]. It is estimated that 1.4–4.7% of middle-aged individuals with diabetes experience a cardiovascular event annually [44,45]. Obese individuals with T2DM exhibit elevated circulating cholesterol levels and are at higher risk for severe arteriosclerosis, characterized by the formation of atherosclerotic plaques within the arterial wall, which narrow the vessel lumen [46]. The resulting restriction in blood flow increases flow velocity and turbulence, further damaging the vascular wall and promoting thrombosis, events that underlie myocardial infarction and stroke [47,48].

Diabetic foot ulcers arise from poor peripheral circulation [49]. Early symptoms of diabetic peripheral neuropathy include hypoesthesia, numbness, and tingling in the lower extremities [50]. Due to sensory loss, injuries to the foot may occur and, because of impaired local circulation, these wounds often fail to heal properly, potentially leading to lower-limb amputation [51].

### 3.1. Pathophysiological Mechanisms Underlying Diabetic Ketoacidosis

DKA is a medical emergency in diabetic patients characterized by plasma glucose levels exceeding 240 mg/dL, resulting from the inhibition of lipogenesis [52]. Consequently, fatty acids fail to enter the citric acid (Krebs) cycle and instead undergo mitochondrial β-oxidation, leading to the formation of ketone bodies [53]. The two primary ketone bodies are acetoacetic acid and β-hydroxybutyric acid, with acetone being the third and least abundant [54]. Ketone bodies are weak acids; however, their excessive accumulation may exceed the compensatory capacity of the body’s acid–base homeostatic mechanisms, leading to severe metabolic acidosis, known as diabetic ketoacidosis, defined by blood pH less than 7.35 and bicarbonate less than 18 mmol/L [55,56]. DKA is further exacerbated by dehydration resulting from osmotic diuresis [55]. If not treated promptly with high-dose insulin therapy and adequate rehydration, DKA can progress to diabetic coma and ultimately death [57]. To compensate for the acidosis, respiration becomes rapid and deep (Kussmaul respiration), promoting increased elimination of carbon dioxide [55]. Additionally, the kidneys contribute to compensatory mechanisms by reabsorbing bicarbonate from the extracellular fluid and synthesizing new bicarbonate ions [58]. However, this renal buffering depletes extracellular bicarbonate reserves [59,60]. Severe DKA develops primarily in cases of poorly controlled diabetes mellitus [61]. When blood pH drops below 7.0, diabetic coma and death may ensue within hours [61]. The clinical signs and symptoms of diabetic ketoacidosis include: (1) elevated blood glucose levels (>240 mg/dL), (2) ketonuria, (3) polydipsia and polyuria, (4) nausea, vomiting, and abdominal pain, (5) dyspnea or labored breathing, (6) a fruity or acetone odor on the breath, (7) flushing, (8) fatigue, (9) dehydration, and (10) loss of consciousness [14,55,60,61].

### 3.2. Pathophysiological Mechanisms of Osmotic Diuresis and Muscle Mass Reduction in T2DM

Under normal physiological conditions, glucose from the renal filtrate is completely reabsorbed by the renal tubules into the peritubular capillaries and reenters systemic circulation [62]. The plasma glucose concentration threshold of 180 mg/dL, known as the renal glucose threshold, represents the saturation point of the glucose transporters that reabsorb glucose from the filtrate into the bloodstream [63]. When plasma glucose levels exceed 180 mg/dL, the filtration of glucose into the renal tubules surpasses the reabsorptive capacity of these transporters, resulting in the excretion of excess glucose in the urine [64]. The elevated glucose concentration within the renal tubules increases the osmotic pressure, thereby reducing tubular water reabsorption by the peritubular capillaries [65]. Consequently, large volumes of water are lost through the urine, leading to dehydration of the extracellular fluid, followed by compensatory dehydration of the intracellular compartment, ultimately causing widespread cellular dehydration [64].

In uncontrolled T2DM, this mechanism manifests clinically as polyuria and intense thirst [66]. The pathophysiology of T2DM also involves inappropriate hypersecretion of glucagon from pancreatic α-cells under hyperglycemic conditions, further elevating plasma glucose levels via enhanced hepatic gluconeogenesis [67]. Simultaneously, glucagon promotes protein catabolism [68]. This reduction in total body protein stores in T2DM patients leads to loss of muscle mass and progressive weakness [68]. Severe muscle wasting may result in quadriplegia and ultimately death [69].

## 4. Antioxidants in T2DM and the Amelioration of Obesity-Associated T2DM

### 4.1. Vitamin E

**Figure 2:**
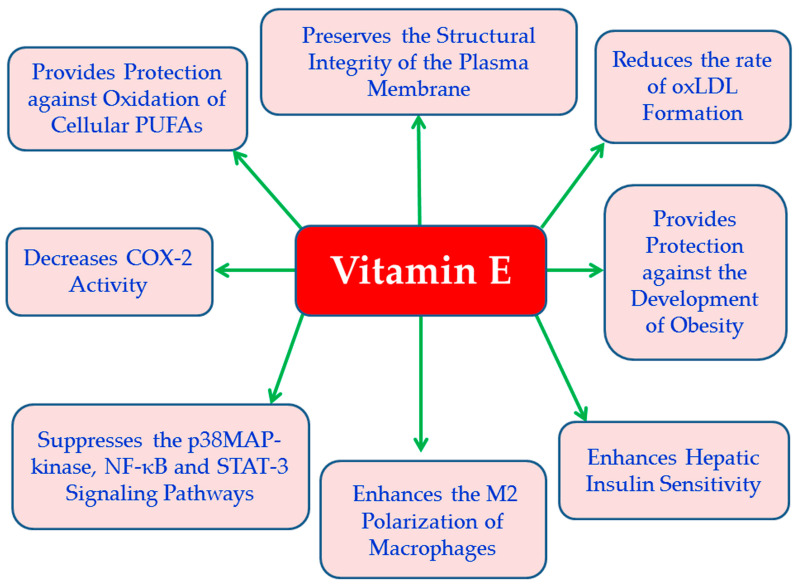
Protective effects of vitamin E against obesity-associated T2DM.

Vitamin E is a group of lipid-soluble substances (tocols) including tocopherols (saturated) and tocotrienols (unsaturated) each with four natural isomers (α-, β-, γ-, and δ-) [70,71,72]. Vitamin E is an important cell membrane component of all tissues and is transported in the body via plasma lipoproteins [73]. All eight forms of vitamin E act as antioxidants when fat undergoes oxidation. In particular, tocopherols and tocotrienols have a hydroxyl (–OH) group on their chromanol ring, which allows them to donate hydrogen atoms to free radicals to prevent the oxidation of tissue polyunsaturated fatty acids (PUFAs) in tissues [74]. Alpha-tocopherol is recognized as the most potent natural source of vitamin E, and it acts as chain-breaking antioxidant in lipoproteins preventing lipid peroxidation and maintaining plasma membrane integrity [75]. Also, alpha-tocopherol reduces LDL oxidation preventing the formation of foam cells and the development of atherosclerosis [74] (Figure 2). Vitamin E supplementation reduces the inflammatory response by reducing the expression of IL-6, TNF-α and C reactive protein [76] (Figure 2). Vitamin E has been found to inhibit the signaling pathways of p38 MAP-kinases [77], NF-κB and STAT-3 [78], and to reduce the COX-2 activity (Figure 2) and the production of eicosanoids [79]. Vitamin E has also shown antidiabetic properties, since it improves insulin sensitivity (alpha-tocopherol) and IR in the liver [80] (Figure 2). The ability of alpha-tocopherol to act as a scavenger of oxLDL seems to be beneficial for the health of patients with metabolic syndrome by assisting to the reduction of the risk of development of T2DM and the prevention of atherosclerosis associated with obesity [80] (Figure 2). In relation to clinical findings, vitamin E has been found in obese animal models to offer anti-obesity effects [81]. Also, in an experimental model of diabetic rats, it was observed that the administration of tocotrienol-rich supplements (200 mg/kg/day) reduced fasting blood glucose levels and levels of oxidative stress markers and ameliorated dyslipidemia [82]. Additionally, in another experimental model of obese mice, it was found that oral administration of γ-tocotrienol supplements (50 mg/kg) significantly reduced fasting blood glucose levels, insulin levels, and proinflammatory cytokine secretion, and enhanced insulin signaling in adipose tissue [83]. At the same time, it has been found that the administration of alpha-tocopherol to obese individuals caused polarization of macrophages towards the anti-inflammatory (M2) phenotype [84] and reduced oxidative damage caused by ROS in T2DM [84].

Although numerous studies support the antioxidant, anti-inflammatory, and metabolic effects of vitamin E, several limitations within the current evidence base should be considered to contextualize these findings. First, many human trials evaluating vitamin E, whether α-tocopherol or tocotrienol-rich preparations, are characterized by small sample sizes, short supplementation periods, and heterogeneous populations, limiting statistical power and reducing confidence in long-term clinical applicability. Additionally, findings across studies remain inconsistent, with some reporting improvements in insulin sensitivity, inflammatory markers, or lipid oxidation, while others show minimal or no effect, particularly when baseline vitamin E status or dietary intake varies considerably. A substantial proportion of mechanistic data demonstrating influence of vitamin E on signaling pathways, oxidative stress markers, or metabolic regulation arises from animal models, which do not fully replicate human lipid metabolism, fat-soluble vitamin absorption, or tocotrienol bioavailability. These models often use high-dose regimens or metabolic conditions that diverge from those observed in humans, complicating translation to clinical practice. Additionally, the range of dose between the studies varies a lot, rendering the deduction of safe conclusions rather challenging. In a similar manner, human trials themselves exhibit marked variability in the form of vitamin E used (α-tocopherol vs. tocotrienols), dosage, duration, baseline antioxidant status, coexisting metabolic disorders, and concurrent therapies, all of which introduce heterogeneity and make cross-study comparisons challenging. Furthermore, the differential bioavailability and physiological actions of tocopherols versus tocotrienols remain only partially understood, raising questions about which form, and at what dose, offers the greatest therapeutic potential for T2DM. Addressing these limitations through larger, longer-term, standardized randomized controlled trials is essential to clarify vitamin E’s role in metabolic regulation and its clinical utility in T2DM management.

### 4.2. Vitamin C

Vitamin C (ascorbic acid) is a compound found both in animals and plants, and functions as a cofactor in many enzymatic reactions, as an enzyme component and as a powerful antioxidant, also affecting other antioxidant enzymes and compounds like vitamin E [85]. Human body is unable to synthesize vitamin C, rendering its supplementation through diet or supplements essential [85]. Several studies have examined the effects of vitamin C in T2DM, with some showing potential benefits, particularly at higher doses, in improving fasting blood glucose and HbA_1c_ levels and reducing IR and OS [86]. Naziroglu et al. evaluated the effects of HRT and vitamin C and E supplementation in postmenopausal women with T2DM, compared to non-diabetic postmenopausal women, and found that the therapy decreased lipid peroxidation and plasma levels of total cholesterol, LDL-cholesterol and triglycerides. Also, there was a significant increase in plasma levels of β-carotene catalase, GPx and reduced glutathione, in postmenopausal women with T2DM, treated with HRT and vitamin C and E combination [87]. Also, Harding et al., in a long-term study in adults of both sexes, examined whether the consumption of fruits and vegetables and the plasma vitamin C levels were negatively associated with the risk of T2DM development [88]. The researchers found a significant association between higher plasma vitamin C levels and a decreased risk of T2DM development, suggesting that the high consumption of foods containing it may prevent T2DM development [88]. In addition, similarly, Rafighi et al. evaluated the outcome of vitamin C and E supplementation for 3 months on T2DM patients, aged 30–60 years with BMI > 25, and found that the consumption of vitamin C, E and C plus E significantly reduced the levels of FBG and HbA1c and increased the levels of SOD and GSH in T2DM patients compared to the placebo group [86].

Although numerous studies have examined the role of vitamin C in T2DM, several limitations within the available evidence should be acknowledged. First, many clinical trials evaluating vitamin C supplementation (as those described above) rely on small sample sizes, short interventions duration, or specific subgroups such as postmenopausal women, which limits the generalizability and statistical power of their findings. Results across studies also remain inconsistent, with some trials reporting improvements in fasting glucose, HbA1c, OS biomarkers, or lipid peroxidation, while others show more modest or no significant metabolic effects particularly at lower doses or in populations without baseline vitamin C deficiency. Moreover, much of the mechanistic insight regarding vitamin’s C antioxidant and insulin-sensitizing effects stems from in vitro experiments and animal models, which do not fully reproduce human redox biology, micronutrient interactions, or the metabolic complexity of chronic hyperglycemia. Human clinical studies themselves exhibit substantial heterogeneity in supplement dosage, duration, baseline vitamin C status, dietary patterns, including fruit and vegetable intake, and concurrent use of antioxidant combinations such as vitamins C and E or hormone therapy. These differences introduce variability that complicates direct comparison across trials and may contribute to conflicting outcomes. Collectively, these limitations underscore the need for larger, longer-term, and better-controlled randomized trials to clarify the dose–response relationship, identify subpopulations most likely to benefit, and establish the clinical relevance of vitamin C supplementation in T2DM management.

### 4.3. N-Acetylcysteine (NAC)

NAC is an N-acetylated form of the amino acid L-cysteine, and it is deacetylated into cysteine, which is then used for the formation of glutathione (GSH) [89]. NAC possesses a wide variety of properties, which may position it as an adjuvant for a broad array of diseases and conditions [89]. NAC reduces ROS through both direct and indirect antioxidant effects [90]. It directly scavenges some free radicals like hydroxyl radicals (•OH), nitrogen dioxide radicals (•NO_2_) and carbon trioxide radicals (CO_3_•^−^) [91]. In addition, it indirectly boosts intracellular antioxidant defenses by serving as a precursor for GSH. The indirect pathway involves the synthesis of GSH, which detoxifies various oxidants and replenishes cellular thiol stores, contributing to redox balance overall [92]. However, the oral bioavailability of GSH is low due to its rapid elimination in the gastrointestinal tract, leading to poor absorption and low blood levels, making traditional oral forms ineffective for therapeutic purposes [93]. Alternative delivery methods using nanoformulations such as SLNs, liposomes and nanoemulsions are being developed for NAC to enhance GSH absorption and efficacy. These lipid-based nanosystems protect NAC from degradation, improve its solubility in the body and facilitate targeted delivery across biological barriers by providing a protective barrier and controlled release. This leads to increased bioavailability, extended half-life, and reduced side effects, improving the therapeutic benefit of NAC for various conditions [94]. Many studies support that NAC treatment in obese mice models shows reduced fat mass, increased insulin sensitivity, and improved glucose and lipid metabolism [95]. Daily NAC treatment for 11 weeks in high-carbohydrate diet-fed rats significantly reduced serum insulin levels and improved insulin sensitivity, preventing the development of IR [96]. Also, NAC treatment was found to increase the phosphorylation of the PI3K/AKT and JNK2/STAT3 signaling pathways [97]. Moreover, NAC has been shown to inhibit lipid accumulation in cultured adipocytes, and to reduce the expression of proteins like HSP70, ACY-1, and transketolase [98]. NAC reduces the release of inflammatory cytokines like TNF-α, IL-6, IL-8, and IL-1β by suppressing NF-κB activation through several mechanisms, including scavenging ROS, inhibiting the IKKβ/NF-κB signaling pathway, and preventing the nuclear translocation of NF-κB [99]. In addition, NAC can reduce triglyceride accumulation in cells by inhibiting adipogenic transcription factors like PPARγ and SREBP-1, as has been demonstrated in various preclinical studies [100]. Furthermore, research indicates that NAC can enhance glucose uptake in adipocytes by influencing the insulin signaling pathway, specifically by modulating the activity of PI3K and IRS-1 [95]. Findings suggest that NAC may prevent obesity-related liver issues, like NASH by normalizing MDA and enhancing the function of antioxidant enzymes [101]. NAC reduces the increased thrombotic tendency in T2DM, boosting platelet GSH levels, which enhances their antioxidant defenses and reduces ROS production. This increased antioxidant capacity helps to counteract the platelet hyperaggregability characteristic of T2DM, potentially making NAC a beneficial therapy for these patients [102]. A growing body of research indicates that NAC supplementation may protect the heart from oxidative damage caused by hyperglycemia, a key factor in diabetic cardiomyopathy. By acting as a potent antioxidant and GSH precursor, NAC helps to reduce ROS in cardiac cells, which in turn can attenuate heart remodeling, improve cardiac function, and potentially prevent myocardial injury [95].

### 4.4. Zinc

Zinc is an essential trace element of living organisms acting as a cofactor of hundreds of enzymes and transcription factors, contributing to DNA and protein synthesis and cellular metabolism and signaling pathways [103]. Zinc also provides molecular structural stability of the cellular membrane [104]. Under normal conditions, zinc binds to MTs in mammals with low affinity, while in oxidative stress zinc is released to exert its antioxidant activity [105]. Zinc is an integral part of the cytoplasmic enzyme superoxide dismutase [106], which promotes the conversion of superoxide radicals into molecular oxygen and H_2_O_2_, reducing ROS production and propagation [107]. Moreover, zinc regulates the expression of GCL, which is involved in the de novo cytoplasmic synthesis of GSH, an effective antioxidant in the body [107]. Zinc supplementation enhances insulin sensitivity by inactivating PTP1B, thereby preventing dephosphorylation of IR [108]. Therefore, zinc improves fasting and postprandial plasma glucose levels [108,109]. Under hyperglycemic conditions, zinc ions increase insulin secretion from pancreatic β-cells, insulin storage capacity, and insulin structural stability, reducing chronic hyperglycemia [109,110]. Thus, patients who cannot be adequately treated with antidiabetic drugs may benefit from zinc supplementation. Patients with T2DM also experience zinc deficiency due to hyperglycemic osmotic diuresis, which causes increased urinary zinc excretion [111]. At the same time, chronic low zinc intake is a risk factor for obesity and T2DM, while its supplementation appears to prevent the onset of metabolic syndrome and T2DM [109]. Wang et al. in a meta-analysis showed that zinc administration improves the glycemic profile of diabetic patients and individuals with risk of T2DM progression [112]. It has also been found that insufficient concentrations of zinc promote the production of IL-1β, IL-2, IL-6 and TNF-α, resulting in oxidative stress [113]. Furthermore, Zn deficiency disrupts cell growth, attenuates intracellular signaling pathways, and enhances the p53 signaling pathway, resulting in apoptosis initiation [114]. However, excessive zinc intake causes copper deficiency and affects the expression of copper-dependent antioxidant enzymes (SOD and ceruloplasmin) [115]. Therefore, the ideal therapeutic regimen of dose and duration of zinc supplementation for the prevention and treatment of T2DM should be determined in adult obese patients [112].

Despite the extensive evidence supporting metabolic and antioxidant role of zinc, several important limitations within the current body of research warrant consideration. Many clinical trials in T2DM involve small sample sizes and relatively short intervention periods, which limit statistical robustness and reduce confidence in long-term clinical relevance. Although meta-analyses often report improvements in glycemic outcomes, inconsistencies remain across individual studies, particularly regarding the extent of fasting glucose reductions, the reliability of effects on insulin sensitivity, and the clinical significance of baseline zinc deficiency. Furthermore, much of the mechanistic understanding of zinc’s antioxidant and insulin-modulating effects derives from animal studies and in vitro models, which do not fully replicate human zinc metabolism, dietary influences, or the complexity of chronic hyperglycemia. Human trials also demonstrate considerable heterogeneity in zinc dosage, chemical form, baseline nutritional status, dietary patterns, and concurrent antidiabetic therapies, making comparisons across studies rather difficult. Additionally, concerns about potential adverse effects, such as copper deficiency and impaired activity of copper-dependent antioxidant enzymes, highlight the need for carefully designed dose–response investigations. Acknowledging these limitations underscores the necessity for larger, longer-duration, and better-standardized randomized controlled trials to determine the optimal therapeutic dose and duration of zinc supplementation in adults with obesity and T2DM.

### 4.5. Alpha-Lipoic Acid

Alpha-lipoic acid (also known as thioctic acid) is an eight-carbon saturated fatty acid [116], which is found in certain foods at low content, such as red meat, spinach, broccoli, potatoes, sweet potatoes, carrots, beets, and yeast [117]. Alpha-lipoic acid is also de novo synthesized in small amounts by the mitochondria of the human body and is an essential coenzyme for several enzymes, such as the 2-oxoglutarate dehydrogenase complex, the branched-chain oxoacetate dehydrogenase complex, and the acetoin dehydrogenase complex [117]. It has a short half-life and bioavailability primarily due to poor solubility, hepatic degradation and instability in the stomach [118]. Alpha-lipoic acid is considered a potent antioxidant modulator of signaling pathways related to inflammation, scavenging ROS and regenerating other antioxidants such as GSH, vitamin C, vitamin E and coenzyme Q10 [119,120]. Alpha-lipoic acid supplementation in diabetic patients seems to significantly reduce the risk of obesity and its complications, exerting its effects by inhibiting hepatic gluconeogenesis and enhancing peripheral glucose uptake into peripheral cells [121]. Ansar et al., in an eight-week randomized double-blind, placebo-controlled trial, found that daily administration of 300 mg alpha-lipoic acid in T2DM patients significantly reduced fasting and postprandial blood glucose levels and IR [122]. Also, Zhang et al. in a 2-week randomized, double-blind, placebo-controlled trial in 22 obese patients found that the intravenous administration of 600 mg alpha-lipoic acid improved insulin sensitivity and plasma lipid profile possibly by ameliorating OS and chronic inflammation [123]. Moreover, in a 20-week double-blind, placebo-controlled trial, in 360 obese patients, was found that *per os* administration of 1800 mg/day alpha-lipoic acid led to a modest weight loss [124]. The positive effects of α-lipoic acid supplementation in the regulation of glucose and lipid metabolism, the reduction in energy intake, the increase in energy expenditure and the loss of body weight possibly seem to be associated with the activation of AMPK [125]. Haghighatdoost et al. in a meta-analysis of randomized controlled trials showed that alpha-lipoic acid supplementation in obese patients for more than 8 weeks appeared to be associated with a decrease in leptin levels and an increase in adiponectin levels [126]. The increased levels of adiponectin after alpha-lipoic acid administration seem to be, at least partly, due to the activation of AMPK and PPAR-γ [127].

Although the available evidence suggests beneficial metabolic and anti-inflammatory effects of α-lipoic acid, several limitations within the current body of research warrant acknowledgement. Many of the cited clinical trials are based on small sample sizes, for example, studies with 22 to 50 participants and brief intervention periods of 2 to 8 weeks, which limits statistical power and reduces confidence in the generalizability of observed improvements in insulin sensitivity, lipid parameters, or inflammatory markers. Furthermore, while some randomized trials and meta-analyses report favorable outcomes, there remain inconsistencies across studies, including variability in the magnitude of glycemic improvements, weight loss effects, and adipokine responses, raising questions about dose-dependence and long-term sustainability. Another consideration is that much of the mechanistic understanding of α-lipoic acid—including its roles in oxidative stress modulation, AMPK activation, and adiponectin regulation—is derived from animal models and in vitro studies, which cannot fully replicate the physiologic and metabolic complexity of individuals with T2DM or obesity. In addition, human studies differ widely in dosage (300–1800 mg/day orally, or IV administration), formulation, study duration, participant characteristics, and concurrent lifestyle or pharmacologic interventions, all of which contribute to substantial heterogeneity and complicate direct comparison of outcomes. Recognizing these limitations would provide a more balanced interpretation of the literature and highlight the need for larger, longer-term, and methodologically standardized randomized controlled trials to more definitively characterize the therapeutic role of α-lipoic acid in T2DM management.

### 4.6. L-Carnitine

Carnitine occurs as two isomeric forms (D and L). D-carnitine is not naturally found in the body, but is only present in some synthetic preparations [128]. In animal models, D-carnitine supplementation has been linked to liver inflammation, OS, and apoptosis [129]. L-carnitine (levo-carnitine) is the biologically active form of carnitines. It is a water-soluble quaternary amine that is vital for energy production, as it transports FFAs into the mitochondria for their conversion to acetyl-CoA via β-oxidation. Then, acetyl-CoA enters the Krebs cycle and through a series of reactions leads to the production of ATP, which provides energy to the cells [117]. Dietary sources of L-carnitine include red meat, fish, poultry, eggs, dairy products, soy, nuts, and seeds [117]. However, vegetables, fruits and grains contain very low amounts of L-carnitine [129]. L-carnitine supplementation has been shown to significantly reduce OS and increase the activity of antioxidant enzymes [117]. A meta-analysis by Fathizadeh et al. revealed that L-carnitine significantly reduced levels of CRP, IL-6, TNF-α, and MDA, and increased SOD [130]. In addition, L-carnitine supplementation significantly reduces body weight [131]. Also, L-carnitine induces beneficial changes in the glycemic and lipid profiles of patients with type II diabetes mellitus who follow a Mediterranean diet in addition to their treatment [132]. While the available evidence on L-carnitine is encouraging, several important limitations should be acknowledged. First, many of the trials assessing L-carnitine’s metabolic and anti-inflammatory effects, including those summarized in meta-analyses, are based on relatively small sample sizes, which may limit the statistical power to detect clinically meaningful changes in glycemic or lipid parameters. Moreover, although meta-analytic findings indicate improvements in inflammatory markers such as CRP, IL-6, TNF-α, and MDA, there remain inconsistencies across individual studies, particularly regarding the magnitude and durability of these effects, as well as their relevance to long-term T2DM outcomes. Another limitation is the heavy reliance on animal models, which, although mechanistically informative, do not fully replicate the metabolic complexity, comorbidities, and lifestyle factors seen in human T2DM; findings such as the pro-oxidative effects of D-carnitine in animals may not translate directly to clinical settings. Additionally, human intervention studies vary considerably in terms of dosage (ranging from low supplemental levels to pharmacological doses), duration (weeks vs. months), participant characteristics (age, disease stage, medication use), and concurrent dietary patterns. This heterogeneity makes it difficult to draw firm conclusions regarding optimal dosing, target populations, or expected clinical benefit.

### 4.7. Coenzyme Q_10_ (CoQ10)

CoQ10, or ubiquinone, is a fat-soluble benzoquinone with a side chain of 10 isoprenoid units, found in almost all human and animal cells, synthesized endogenously from precursors derived from both phenylalanine (for the benzoquinone ring) and mevalonic acid (for the isoprenoid side chain) [133]. Coenzyme Q10 exists in 2 forms: the oxidized form (ubiquinones, oCoQ10) and the reduced form (ubiquinol, rCoQ10) [133]. Within cells, ubiquinol and ubiquinone are interconverted between oxidized and reduced forms [134]. CoQ10 is involved in the mitochondrial electron transport chain, for the synthesis of ATP and therefore has a key role in cellular energy supply [135]. A small increase in the concentration of coenzyme Q10 in mitochondrial membranes can increase the mitochondrial respiration [136]. Primary dietary sources of CoQ10 include beef and pork, organ meats (such as heart, kidneys and liver), poultry, oily fish (such as salmon and tuna), olive oil, soybeans, grape seeds, whole grains, nuts and most dairy products (in smaller amounts) [137]. CoQ10 absorption from the diet occurs mainly in the small intestine and is best absorbed by taking a meal containing oil or foods rich in fat and is then transported to the liver via lipoprotein complexation [138]. Ubiquinol supplements offer superior bioavailability compared to ubiquinone probably due to more efficient absorption and incorporation into mixed micelles in the intestine [139]. Also, intestinal absorption of CoQ10 varies significantly between individuals [134]. CoQ10 as a dietary supplement is relatively safe, but approximately 1% of the population may experience side effects such as headache, stomach ache, diarrhea, loss of appetite, dizziness, irritability, itching, skin rash, and nausea [140]. CoQ10 possesses significant antioxidant and anti-inflammatory properties [141], protecting cellular components like lipids, proteins, and DNA from oxidative damage [142], and also modulating inflammatory gene expression to reduce pro-inflammatory cytokines, such as TNF-α and IL-6 [143]. However, CoQ10 supplementation does not appear to improve body weight, BMI and waist circumference [144], but seems to have beneficial effects on glycemic control, lipid profile and blood pressure in patients with T2DM [145]. The beneficial effects of CoQ10 on lipids may be due to increased expression of PPAR-γ gene, which produces a nuclear receptor protein that regulates lipid metabolism and inflammation [146,147]. In addition, CoQ10 supplementation regulates hepatic lipid metabolism by the activation of the AMPK pathway [148]. Moreover, CoQ10 supplementation suppresses the LOX-1 receptor and reduces the ROS production via inhibition of the NADPH oxidase and other inflammatory pathways [149]. Despite these promising findings, several limitations in the current literature warrant attention. Many mechanistic studies rely on in vitro or animal models that may not accurately reflect human CoQ10 pharmacokinetics, tissue distribution, or chronic disease progression. Rodent models often use supraphysiological doses that do not correspond to typical human supplementation patterns. Clinical trials examining CoQ10′s effects on T2DM are variable in sample size, duration, and methodology, with many involving relatively small cohorts that limit statistical power. Moreover, heterogeneity in supplement formulations (ubiquinol vs. ubiquinone), dosing regimens, baseline CoQ10 status, and concurrent medications may contribute to inconsistent findings across studies. Additionally, interindividual variability in CoQ10 absorption further complicates interpretation of efficacy.

### 4.8. Superoxide Dismutases (SODs)

SODs are crucial endogenous metalloenzymes that protect cells by converting superoxide radicals (O_2_•^−^) into hydrogen peroxide (H_2_O_2_) and molecular oxygen (O_2_) [142]. Then, other endogenous antioxidant enzymes such as catalase, GPx and Prx eliminate hydrogen peroxide (H_2_O_2_) reducing it to water (H_2_O) [142]. On the other hand, under specific conditions SODs can indirectly act as prooxidant by accumulating hydrogen peroxide (H_2_O_2_), which in the presence of ferrous ions (Fe^2+^) undergoes the Fenton reaction. This reaction produces high reactive hydroxyl radicals (•OH), which are toxic ROS that cause cellular damage [142]. In mammals, the three SOD isoforms are SOD1 (copper-zinc SOD) located in the cytoplasm; SOD2 (manganese SOD) located in mitochondria; and SOD3 (extracellular cooper-zinc SOD), located in the extracellular space. Each isoform is produced by a distinct gene, but requires different metal cofactors for its catalytic activity produced by distinct genes and characterized according to the location they are located and the metal they catalyze [150]. TNF-α is a potent activator of SOD2 contributing to its immune-modulatory effects [151]. Inflammatory mediators like IL-1, IL-4, IL-6, and TNF-α activate SOD2, which is induced by its binding sites for transcription factors such as NF-κB, NF-1, and C/EBPs within the SOD2 gene [151]. Evidence indicates that SODs and their mimetics can improve the obesity phenotype by reducing OS and inflammation, which are key features of obesity [152]. In an in vivo study, mice fed a high-fat diet and supplemented with the antioxidant Tempol experienced significantly greater body weight loss and reduced lipid accumulation compared to control mice, which was linked to alterations in the gut microbiome and bile acid composition [153]. Mice on HFDs receiving hydrodynamic injections of SOD3 plasmids showed improved metabolic health markers, including reduced body weight, blood levels of triglycerides and cholesterol, along with better glucose tolerance and insulin sensitivity, compared to control mice. Additionally, the metabolic improvement was associated with higher mRNA levels of key genes involved in energy expenditure and breakdown, such as CPT1α, CPT1β, PGC1α, PGC1β, and UCP2 [154]. Also, an in vivo study using nanoparticulated SOD on mice with a HFD demonstrated improvements in lipid metabolism, specifically by reducing serum triglyceride levels, decreasing liver triglyceride content, and mitigating hepatic lipid accumulation, which are key indicators of metabolic dysfunction associated with obesity [155]. Coudriet et al. found that treating T2DM-induced HFD in mice with a manganese porphyrin SOD mimetic improved mitochondrial function, glucose tolerance and IR by stabilizing mitochondrial membranes and enhancing aconitase activity. Additionally, the MnP treatment improved liver function and reduced hepatic steatosis [156]. Despite these promising findings, much of the current evidence comes from preclinical animal models, which, while mechanistically informative, do not fully capture the complexity, heterogeneity, and chronic progression of human T2DM. Sample sizes in many of these studies are small, limiting statistical power and increasing the risk of overstating treatment effects. Moreover, the metabolic outcomes reported across studies can vary, with some models demonstrating improvements in weight or lipid profiles, while others report predominantly biochemical or mitochondrial benefits without clear effects on glucose homeostasis. Additionally, human clinical data on SOD enzymes or SOD mimetics in metabolic diseases remain scarce, and available interventions differ substantially in formulation, delivery method, and pharmacokinetic behavior, making it difficult to extrapolate or generalize findings.

### 4.9. Catechins

**Figure 3:**
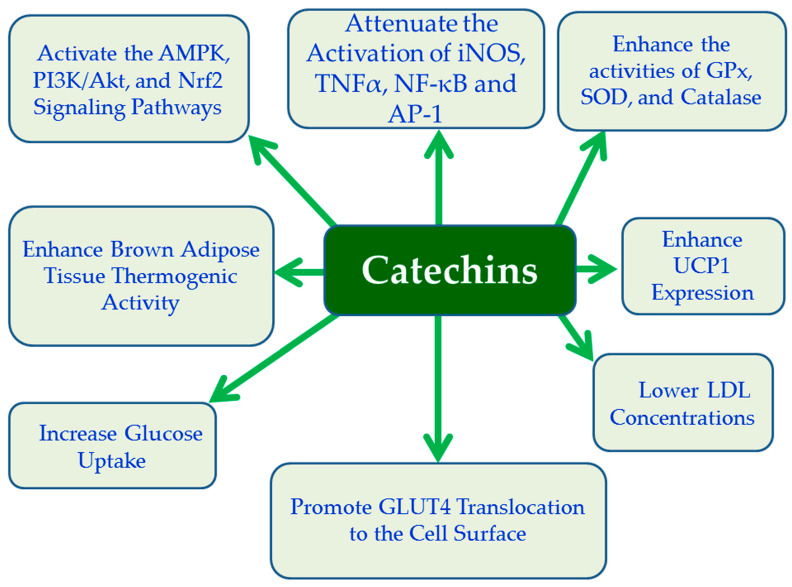
Protective effects of catechins against obesity-associated T2DM.

Catechins, including EGCG, are plant compounds classified as flavanols, a subgroup of flavonoids, known for their potent antioxidant, anti-inflammatory, and therapeutic properties. Found primarily in green tea, cocoa, red wine, apples, and berries, catechins act by scavenging free radicals, inhibiting OS, and modulating signaling pathways [153]. EGCG exhibits antioxidant effects by increasing the activity of the antioxidant enzymes GPx, SOD, catalase and anti-inflammatory effects, by decreasing the activation of inflammatory markers like iNOS, TNFα, NF-κB, and AP-1, which contribute to cellular damage and inflammatory response [153,157] (Figure 3). Moreover, EGCG activates the Nrf2 pathway by stimulating the PI3K signaling cascade, which leads to beneficial effects in ameliorating various diabetic complications, such as diabetic nephropathy through the reduction of oxidative damage, inflammation, and fibrosis [158] (Figure 3). Furthermore, EGCG acts like insulin promoting glucose uptake by triggering the translocation of GLUT4 from inside the cell to its surface, a process that requires PI3K/Akt pathway and activation of AMPK [159] (Figure 3). In parallel, EGCG supplementation enhances BAT thermogenesis by activating AMPK pathway and increasing UCP1, thereby promoting heat production, a process critical for energy expenditure and anti-obesity effects [160] (Figure 3). Accumulating evidence suggests that EGCG supplementation, often in the form of green tea extract, improves endothelial function and has significant beneficial effects in reducing SBP by reducing the secretion of vasoconstrictor substances like ET-1 and increasing the production of NO [161]. Also, consumption of green tea EGCG can reduce LDL cholesterol levels [162], accentuating the potential positive effects of EGCG on metabolic imbalances provoked by oxidative stress and inflammatory conditions (Figure 3). Despite substantial mechanistic evidence, human studies are scarce and show mixed results due to differences in EGCG dosing, purity of green tea extracts, caffeine content, and interindividual variability in catechin metabolism.

### 4.10. Quercetin

Quercetin (3,5,7,3′,4′-pentahydroxyflavone) is a natural yellow flavonol [163]. Quercetin is found in many fruits such as cherries, berries and apples; vegetables such as onions, capers, cabbage, red lettuce leaves, and broccoli; nuts; and beverages such as green tea, coffee and red wine [164]. Also, quercetin is found in various medicinal plants such as Acanthopanax seticosus (Siberian ginseng), momordica charantia (bitter melon), and brassica rapa (turnip) and has been used in traditional medicine [163]. It is completely soluble in lipids and alcohol, but not in water, which limits its absorption from the digestive system [163]. Also, quercetin has low oral bioavailability because it is extensively metabolized into inactive forms that are quickly excreted [165]. This low oral bioavailability is a major challenge for its clinical use, but methods like nanoformulations are being explored to help improve its uptake and delivery to the target site [166]. Another factor contributing to its low bioavailability includes its rapid clearance from the body [164]. Quercetin is absorbed primarily in the small intestine, with some evidence suggesting that certain forms, like quercetin glucosides, can be transported by the sodium-glucose cotransporter 1 (SGLT1) [165]. Absorption in the stomach is limited, and its poor solubility can hinder its absorption without fat parallel intake [165]. Quercetin absorbed by enterocytes is rapidly conjugated with glucuronic acid and/or sulfate by enzymes during first-pass metabolism, which significantly reduces the amount of free quercetin available in the bloodstream. These reactions convert quercetin into more water-soluble forms like quercetin-3-O-glucuronide and quercetin-3′-O-sulfate [167]. During phase II metabolism, quercetin in the intestine, kidneys and liver undergoes glucuronidation, sulfation, and methylation, leading to excretion in bile, urine and feces. During this process, enzymes like UGTs, SULTs, and COMTs add hydrophilic groups to quercetin, making it more water-soluble and easier to be excreted from the body [165]. Quercetin has a wide range of pharmacological properties, including antioxidant, anti-inflammatory, antihypertensive, and vasodilatory effects, which contribute to its potential application in treating conditions like obesity, diabetes and cardiovascular diseases. It is also being studied for its anticancer and neuroprotective effects [168]. Quercetin’s antioxidant activity involves activating the Keap1-Nrf2 pathway. Quercetin assists to the disruption of the KEAP1-NRF2 interaction, allowing NRF2 to translocate to the nucleus and activate this protective antioxidant response [169]. In addition, quercetin can reduce serum levels of MDA, increasing the antioxidant potential via the reduction of lipid peroxidation [170]. Furthermore, quercetin reduces inflammation by suppressing the release of pro-inflammatory cytokines like IL-1β, IL-6, and TNF-α, and by lowering the expression of chemokines such as MCP-1 by inhibiting the NF-κB pathway [171]. Data suggests that quercetin prevents lipogenesis by decreasing the production of new fat molecules and promoting their breakdown. This is achieved by suppressing key enzymes involved in fat synthesis, such as ACACA and FASN, and transcription factors like SREBP-1c as well. It also simultaneously promotes fat breakdown by up-regulating enzymes involved in fatty acid oxidation. This dual action leads to a reduction in overall body fat accumulation [172]. Also, quercetin can modulate the insulin signaling pathway to further decrease fat and triglyceride accumulation in hepatic cells, therefore providing protection from NAFLD development [173]. Quercetin has shown significant antidiabetic potential by inhibiting intestinal glucose absorption, improving insulin secretion and sensitivity, and boosting glucose uptake in tissues like the liver and skeletal muscle. It also protects pancreatic cells from damage and reduces inflammation, although its low bioavailability is a hurdle for effective human application [163]. Research shows that quercetin, in rats with STZ induced diabetes, lowered plasma glucose levels and improved glucose tolerance by regenerating pancreatic islets, increasing insulin secretion and improving antioxidant defense. It also enhances glucose uptake by activating insulin signaling molecules, such as PI3K and IRS-1, which leads to a significant reduction in plasma glucose levels and improved glucose tolerance [163]. In addition, a 12-week treatment of quercetin in STZ-induced diabetic rats does promote hepatic glycogen synthesis and lower blood glucose levels by activating the AKT and SIRT1 signaling pathways [174]. This is achieved because quercetin activation of SIRT1 leads to subsequent activation of the AKT pathway, which can improve glucose and lipid metabolism [174]. In hepatocytes, quercetin activates AMPK, which in turn inhibits the expression of gluconeogenic enzymes [175]. Moreover, in skeletal muscle cells, quercetin activates PI3K/Akt and AMPK pathways, which promote the translocation of the GLUT4 transporter and the glucose uptake. This mechanism is similar to some anti-diabetic drugs and suggests that quercetin enhances glucose metabolism by improving also insulin sensitivity [176]. However, at clinical level, daily oral quercetin supplementation at 250 mg for 8 weeks did not significantly alter glycemic control or lipid profiles, but it improved the antioxidant status [177]. However, quercetin intake at daily doses of 500 mg or more for a period of at least 8 weeks significantly reduced fasting plasma glucose levels in individuals with metabolic disorders. This effect is likely due to quercetin’s ability to improve insulin sensitivity and stimulate glucose uptake [178]. Quercetin also protects against complications of diabetes mellitus by lowering blood glucose, reducing oxidative stress, and exerting anti-inflammatory effects [179]. A dose of 150 mg/kg of quercetin has been shown to improve diabetic retinopathy in streptozotocin-induced rats by activating the HO-1 pathway, which helps counteract OS, inflammation and neovascularization associated with the disease [180]. In addition, quercetin inhibits the SphK1-S1P pathway, which is involved in development of renal fibrosis in cases of diabetic kidneys. This inhibition can protect against diabetic complications, leading to reduced renal fibrosis [181]. Furthermore, research shows that quercetin can increase cardioprotection in rats with streptozotocin-induced diabetes by improving endothelial function. This is achieved by reducing OS, which in turn increases the production of NO and protects against aortic damage caused by the diabetes [182]. Also, it has been found that quercetin supplementation in T2DM women significantly reduces systolic blood pressure [183]. Despite promising mechanistic and preclinical results, several limitations temper the strength of current evidence. Much of the data derives from in vitro studies and STZ-induced diabetic rodent models, which replicate only selected features of human T2DM and often use doses far exceeding typical human intake. Human clinical trials vary considerably in quercetin dose, formulation, duration, and participant populations, contributing to inconsistent outcomes—particularly regarding glycemic control, where some studies report no significant changes despite biochemical improvements. Many trials use modest sample sizes and short intervention periods, limiting their statistical power and relevance to chronic metabolic disease. The extensive metabolism of quercetin and the use of heterogeneous formulations also complicate the comparison and interpretation of results across studies. These limitations underscore the need for larger, well-controlled, formulation-standardized human trials to clarify quercetin’s therapeutic potential in T2DM.

### 4.11. Curcumin

Curcumin is the main active compound from the turmeric plant (*Curcuma* *longa*) and is a pleiotropic polyphenol, including strong antioxidant and anti-inflammatory properties [184]. The low bioavailability of curcumin is related to its low water solubility and poor permeability through the intestine, and rapid metabolism and clearance from the body [185]. Curcumin reduces inflammation by inhibiting key signaling pathways like NF-κB, MAPK, and JAK/STAT, which decrease the production of cytokines as TNF-α and INF-γ, and chemokines like RANTES [184]. At the same time, curcumin functions as a metal chelator and directly scavenges ROS. It also indirectly boosts antioxidant defenses by increasing the activity of enzymes as is SOD and catalase, which reduces OS markers (MDA) and increases TAC [184]. Curcumin improves insulin sensitivity by inhibiting pro-inflammatory signals like ERK/JNK phosphorylation and activating PI3K-Akt-GSK3B pathway, which promotes glucose uptake [186]. Also, curcumin promotes insulin sensitivity and redox homeostasis by regulating Keap1 expression to activate Nrf2 [184]. In addition, it improves hyperglycemia [187] and hyperlipidemia by reducing triglycerides, total cholesterol, and LDL-c levels, and increasing HDL-c levels [188]. Moreover, it enhances weight loss through metabolic regulatory, anti-inflammatory and antioxidant properties [187,189]. Ιn vitro data suggest that curcumin can promote the “browning” of white adipocytes, leading to enhanced lipolysis, by increasing the levels of hormone-sensitive lipase and p-acyl-CoA carboxylase [189]. Accumulating evidence suggests that curcumin can modulate pathways involved in metabolic diseases by suppressing the inflammatory NF-κB pathway and its regulators IKK and JNK [187,190]. Curcumin regulates signaling pathways that target inflammatory mediators in diabetes mellitus, improves beta-cell function, and reduces IR through its antioxidant and anti-inflammatory properties [191]. In addition, curcumin can inhibit enzymes such as α-glucosidase and aldose reductase, which are linked to diabetes complications [186]. Panahi et al. found that providing 1000 mg of curcuminoids daily for 12 weeks to patients with type 2 diabetes promotes serum TAC and SOD activity and reduces MDA levels [192]. A systematic review by Matron et al., found that curcumin supplementation significantly reduced BMI, HbA1c and fasting blood glucose levels [193], whilst curcumin-based nanoparticles are also being investigated for their potential therapeutic properties in T2DM, in order to enhance its solubility, bioavailability and transport across biological membranes [184]. Most mechanistic evidence derives from in vitro experiments or animal models that use high doses of curcumin not feasible in humans. Human clinical trials vary widely in formulation (standard extract vs. enhanced-bioavailability complexes), dose, duration, and participant characteristics, leading to inconsistent outcomes across studies. Systematic reviews also note heterogeneity and publication bias, and not all studies observe significant glycemic improvements. Collectively, these limitations highlight the need for larger, longer-duration, well-controlled trials using standardized curcumin formulations to establish its therapeutic relevance in T2DM.

### 4.12. Resveratrol (RSV)

RSV (3,4′,5-trihydroxystilbene) is a polyphenol found in foods like red wine, red grapes, berries, apples, plums, blueberries, peanuts, and chocolate [194]. RSV is associated with a range of health benefits, such as antioxidant, and anti-aging effects, which may help in the prevention or management of conditions like T2DM, CVDs, certain cancers, and neurodegenerative disorders [184]. However, RSV’s rapid metabolism in the small intestine and by the gut bacteria, along with its poor water solubility, leads to low plasma bioavailability, which can limit its effectiveness [195]. RSV modifies the gut microbiota by increasing beneficial bacteria like Bacteroidetes and Bifidobacterium, while reducing opportunistic pathogens. This modulation of the gut microbiome is associated with benefits for metabolic and cardiovascular health, including a reduction in levels of TMAO [196]. RSV action involves the inhibition of PPARγ and activation of SIRT1 leading to a decrease in BMI by reducing lipogenesis and increasing lipolysis [197]. RSV provides potent antioxidant effects in diabetic models by activating the Keap1/Nrf2 pathway, increasing Nrf2 protein levels and the expression of antioxidant genes such as HO-1 and GST. This activation process reduces accumulated ROS and is a key mechanism by which RSV protects against OS and other diabetes-associated complications [197,198]. Moreover, RSV has demonstrated anti-inflammatory effects in diabetic models, primarily by inhibiting the NF-κB pathway. This action decreases pro-inflammatory cytokines like TNF-α, IL-1β, and IL-6, which in turn improves insulin signaling and protects insulin-producing cells from damage in T2DM [198]. The anti-inflammatory effects of RSV are, in part, mediated by its activation of the SIRT1 enzyme, which then deactivates NF-κB by binding to its p65 subunit [199]. Inactivating NF-κB halts the transcription of genes related to inflammatory molecules production like the adhesion molecules ICAM-1 and VCAM-1 [200]. In parallel, RSV protects pancreatic β-cells via the NF-κB signaling cascade and pro-apoptotic signals regulation, which improve β-cell survival [198]. In addition, RSV has potent antidiabetic properties by activating the AMPK/SIRT1 pathway to improve insulin sensitivity in tissues like the liver and muscle. This activation enhances insulin signaling, leading to improved glucose uptake and metabolism [198]. The activation of SIRT1 and AMPK by resveratrol in turn activates FOXO1 and PGC-1α. This activation leads to improved mitochondrial function and protects against metabolic disorders like T2DM [198]. At the same time, RSV suppresses the negative regulator PTP1B, which allows the IRS-1/PI3K/AKT pathway to remain active, thereby promoting insulin sensitivity and glucose uptake [198]. RSV can improve glucose metabolism in muscle cells through the promotion of the Akt pathway, which triggers the translocation of the GLUT4 protein to the cell membrane [198]. A meta-analysis by Abdelhaleem et al. of 17 RCTs with 871 patients with T2DM concluded that supplementation of RSV at doses of 500 mg or more significantly improved glycemic control and cardiometabolic parameters compared to placebo. Specifically, it led to significant improvements in FBG and HbA1c levels, and TC levels and reduced systolic blood pressure [201]. Zeraattalab-Motlagh et al. found that RSV supplementation showed, at least partly, beneficial effects in patients with T2DM, MetS, and NAFLD, such as improvement of lipid profiles, glycemic control, and inflammatory markers. However, the study concluded that the overall evidence does not support the usage of RSV for the management of cardiometabolic risk factors in these patients groups due to the trivial magnitude of effects, low certainty of evidence, and limited trial numbers for most outcomes. A specific exception was the short-term (<12 weeks) improvement in HbA1c, which was clinically significant but requires further research given the limitations [202]. Despite promising mechanistic insight from in vitro and animal models, many of these studies use high RSV doses that exceed physiologically achievable levels in humans, limiting translational relevance. On the other hand, human trials remain heterogeneous, with variability in sample size, study design, RSV dose, duration, and population characteristics, leading to inconsistent findings across studies.

### 4.13. Lycopene

Carotenoids are a group of over 700 naturally occurring compounds synthesized by plants, algae and microorganisms like fungi, and bacteria that provide yellow, orange, and red colors [203,204]. Lycopene, also known as psi-carotene, is a fat-soluble pigment primarily found in tomatoes [205]. Also, other foods like red guava, papaya, watermelon, pink grapefruit, red grapes, apricots, rose hips, eggplant and even potatoes contain lycopene [203]. Moreover, lycopene can be found in certain algae, and fungi [203]. Lycopene has both antioxidant and anti-inflammatory properties that protect the body from cellular damage [206]. Its antioxidant role involves neutralizing of harmful ROS via electron transfer, where lycopene acts as an electron donor [207,208]. The anti-inflammatory lycopene effects come from its ability to inhibit pro-inflammatory cytokines like TNF-α, IL-1β and IL-6, and block signaling pathways like the NF-κB pathway, which decreases inflammation in both adipocytes and macrophages [209,210]. Lycopene seems to prevent obesity-related inflammation by shifting adipose tissue macrophages (ATM) from M1 state to M2 state [211]. Lycopene consumption contributes to obesity in animal studies. Particularly, in animal models, lycopene has been shown to reduce blood lipid levels, prevent weight gain, and decrease liver fat accumulation by improving overall lipid metabolism [209]. Also, a 12-week study on Swiss albino mice showed that lycopene counteracts the effects of a HFD by preventing weight gain, reducing fat in adipose tissue, and improving IR. These are achieved by decreasing total triglyceride levels, improving liver function related to glucose and lipids, and boosting glucose clearance and insulin sensitivity [212]. In addition, a study on C57BL/6J mice found that lycopene improved metabolic health by promoting lipolysis and improving glucose control, while also reducing fat-related markers like triglycerides, NEFA, and HOMA-IR. Lycopene also reduces inflammation and adipocyte hypertrophy and inhibits genes linked to lipid storage, suggesting a multifaceted beneficial effect on obesity-related metabolic dysfunction [213]. Moreover, in vivo lycopene reduces fat accumulation intervening in lipogenesis, thermogenetic and mitochondrial functional genes (Fas, ACACA, PPARγ, PGC1α, Prdm16, UCPs, Sirt1). It also prevents lipid buildup caused by autophagy by down-regulating autophagy-related genes (Atg7, Atg14, P62, Lc3, Beclin) [214]. Furthermore, a study on Wister rats showed it could inhibit obesity derived complications by preventing body and liver weight gain, and improving blood lipid profile (affecting serum levels of cholesterol, LDL, TGs, and Apo-B), while raising HDL serum levels. Also, lycopene can mitigate liver damage from obesity by reducing OS, inflammation, and fibrosis through antioxidant and anti-inflammatory actions. At the same time, lycopene can prevent cardiac complications by reducing LDH and creatine kinase (CK) levels [215].

Lycopene acts against diabetes and its complications through multiple mechanisms, including improving antioxidant defenses, and reducing oxidative stress markers like MDA and inflammatory markers like CRP. It also improves glucose control by reducing markers such as HbA1c and positively influences cell survival pathways by modulating proteins involved in apoptosis and cell death like RAGE, NF-қB, Bax, Bcl-Xl, and Bcl-2 [216]. It has been found that the long-term intake of cooked tomatoes (around 200 g per day) can improve antioxidant status in patients with T2DM by increasing levels of antioxidant enzymes like SOD, GSH, GPx, and GR and reducing lipid peroxidation (MDA levels) after 30 days [217]. Moreover, Shidfar et al. found that 32 patients who received 200 g raw tomato daily for 8 weeks showed significant decreases in both systolic and diastolic pressure, along with a significant increase in ApoA-1. These findings suggest that daily tomato consumption may be beneficial for the managements of some cardiovascular risk factors in patients with T2DM [218]. Also, Singh et al. conducted a 3-month study on subjects with T2DM and found that daily lycopene supplementation of 4 mg improved their oxidative-antioxidant status. Participants receiving lycopene showed significant increases in antioxidant enzymes (SOD, GPx, GR) and reduced levels of MDA, along with reduced XOD levels, compared to the non-lycopene group [219]. In parallel, Guo et al. showed that lycopene increases the expression of the enzyme HO-1 in the kidneys of diabetic individuals, protecting against diabetic kidney damage, by reducing oxidative stress and inflammation. This increase in HO-1 expression maintains renal metabolic homeostasis by mitigating damage from conditions like diabetic nephropathy [220]. In addition, Ozmen et al. showed that lycopene treatment in STZ-induced diabetic rats can alleviate diabetes-related pancreatic damage, reduce blood and urine glucose levels, and increase serum insulin levels [221]. Studies on albino rats show that lycopene has potential to prevent T2DM and improve diabetic neuropathy by acting as an antioxidant and improving metabolic health. It reduced OS, as seen by the increased levels of antioxidant enzymes like SOD and GSH-Px and decreased MDA levels. Lycopene also improves glycolipid metabolism by lowering blood glucose, TG, TC, LDL, and GHb, while raising HDL and insulin levels. Furthermore, it can inhibit inflammation by decreasing TNF-α and NO generation [222]. Furthermore, Gao et al. found in 1978 pregnant women a statistically significant inverse relationship between lycopene consumption and the risk of GDM, especially in the first trimester of the pregnancy [223]. Despite these promising findings, several limitations must be acknowledged. Most mechanistic insights derive from animal models, often using supra-physiological lycopene doses, limiting translational relevance. Human studies frequently involve small sample sizes, short intervention periods, and heterogeneous populations, reducing statistical power and generalizability. Moreover, clinical findings show some inconsistency, particularly regarding glycemic outcomes, likely due to differences in lycopene formulation (dietary vs. supplemental), bioavailability, baseline antioxidant status, and dietary patterns. Additionally, observational associations, such as those regarding GDM risk, may be confounded by overall diet quality and cannot establish causality.

## 5. Conclusions, Potential Applications and Future Perspectives

T2DM can be prevented by maintaining body weight within normal limits and adopting a combination of a healthy diet and regular physical activity. In cases where the prevention of T2DM onset fails, early diagnosis is essential to avoid the development of its complications. Obesity plays a central role in the pathogenesis of T2DM, as it induces a series of disturbances such as extensive expansion of adipose tissue, chronic low-grade inflammation, OS due to the excessive presence of ROS and RNS, dysfunction of the liver and skeletal muscles, pancreatic toxicity resulting in a decreased number of functional β-cells, and dysregulation of the microbiota–gut–brain axis. Insulin signaling, inflammation, OS, and antioxidant defenses constitute a tightly interconnected regulatory network in which dysfunction in any single component amplifies disturbances across the system. Impaired insulin signaling, often triggered by nutrient excess or increased PTEN and MAPK activity, enhances serine phosphorylation of IRS-1 in place of normal tyrosine phosphorylation and diminishes PI3K/AKT pathway activation, thereby increasing the susceptibility of metabolic tissues to inflammatory cytokines. PTEN functions as a pivotal negative regulator of insulin action by dephosphorylating PIP3 to PIP2, attenuating downstream PI3K/AKT signaling and its associated metabolic processes, including glucose uptake, glycogen synthesis, and lipid metabolism [224]. Elevated PTEN expression in adipose tissue, liver, and skeletal muscle has been consistently associated with obesity, insulin resistance, and impaired glucose homeostasis in both animal models and humans [225,226,227]. In T2DM, increased PTEN expression or activity contributes directly to defective insulin signaling and the progression of IR [228]. Consequently, genetic or pharmacological modulation of PTEN or its upstream regulatory pathways may enhance insulin sensitivity, identifying PTEN as a potential therapeutic target in obesity-associated T2DM. Inflammatory mediators such as TNF-α and IL-6 activate stress-responsive kinases, including JNK and p38 MAPK, which further inhibit insulin signaling, while promoting ROS generation through mitochondrial dysfunction and NADPH oxidase activation. Accumulating OS subsequently exacerbates IR by oxidizing key components of the insulin signaling cascade, reducing mitochondrial ATP production, and amplifying inflammatory signaling. ERK1/2, activated via the Ras–Raf–MEK pathway, primarily mediates mitogenic responses such as cell growth and gene expression, and is increasingly implicated in hyperinsulinemia-driven tissue remodeling. Modulating ERK signaling may therefore represent a therapeutic strategy for mitigating insulin resistance and T2DM progression [229]. Similarly, p38 MAPK regulates stress-responsive transcriptional programs and directly modulates key metabolic processes, including GLUT4 trafficking, inflammatory cytokine production, and cellular insulin sensitivity that are disrupted in obesity and contribute to IR and T2DM development [230,231]. Given its central role in integrating inflammatory signals, regulating glucose metabolism, and coordinating cellular stress responses, p38 MAPK represents a compelling therapeutic target for enhancing insulin sensitivity and slowing the progression of T2DM [232].

**Figure 4:**
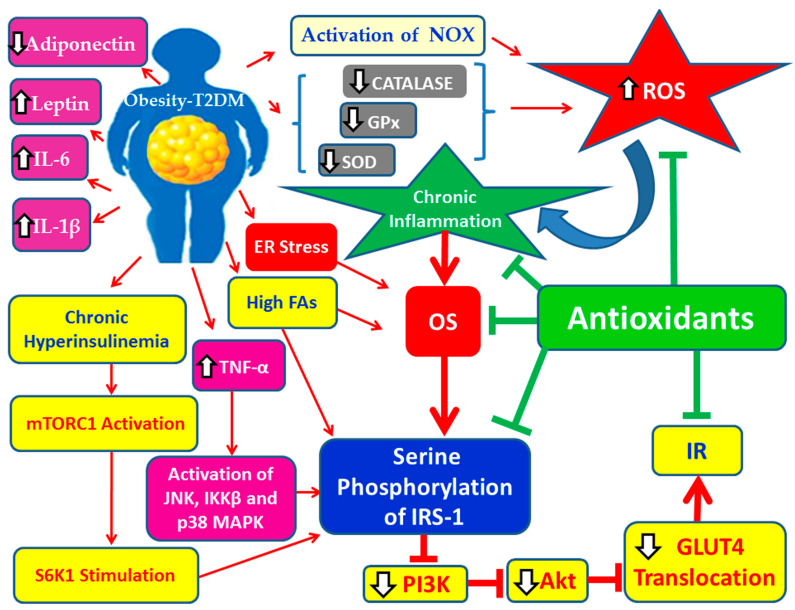
Disruption of insulin signaling by obesity-induced OS and the protective role of antioxidant compounds against OS–mediated IR. Obesity promotes a chronic inflammatory state characterized by elevated pro-inflammatory cytokines such as TNF-α, IL-6, and IL-1β, along with dysregulated adipokines, including increased leptin and reduced adiponectin levels. These inflammatory signals activate stress-responsive kinases such as JNK, IKKβ, and p38 MAPK, while high fatty acid levels, mTOR/S6K1 pathway activation, and hyperinsulinemia further exacerbate metabolic stress. Concurrent ER stress and NOX-mediated ROS production contribute to heightened OS, which is exacerbated by suppression of antioxidant enzyme activity, including catalase, SOD, and GPx. These factors drive serine phosphorylation of IRS-1, leading to impaired insulin signaling and the development of IR. Antioxidant compounds counteract this process by scavenging ROS, reducing OS–induced IRS-1 serine phosphorylation, and preserving insulin signaling integrity.

Hyperinsulinemia itself is a major driver of OS through several complementary mechanisms, including activation of NOX isoforms, mitochondrial dysfunction with excessive ROS generation, suppression of endogenous antioxidant defense systems, and amplification of inflammatory signaling. Chronic elevations in insulin stimulate NOX2 and NOX4 in adipose tissue, skeletal muscle, and vascular cells, resulting in increased production of superoxide anions (O_2_•^−^) [231]. NOX-derived ROS impair insulin action by oxidizing essential intermediates of the PI3K/AKT pathway and promoting serine phosphorylation of IRS-1, thereby attenuating downstream insulin signaling [231]. Concurrently, persistent hyperinsulinemia increases mitochondrial substrate influx—including both glucose and fatty acids—which enhances ETC activity [233,234]. Under nutrient excess, elevated substrate flux promotes electron leakage at complexes I and III, generating additional ROS and progressively compromising mitochondrial function; this mitochondrial stress further aggravates IR [233,234]. Hyperinsulinemia also suppresses the expression of major antioxidant enzymes, including SOD, catalase, and GPx, largely through inhibition of FOXO transcription factors, thereby shifting cellular redox balance toward ROS accumulation [235]. ROS generated from both NOX activation and mitochondrial dysfunction activate stress-responsive kinases such as JNK and p38 MAPK, which subsequently stimulate production of pro-inflammatory cytokines like TNF-α, and IL-6. These cytokines further impair insulin signaling and perpetuate the cycle of OS, inflammation, and metabolic dysfunction [231,236]. Figure 4 summarizes cumulatively the mechanisms by which obesity-induced OS disrupts insulin signaling and highlights how antioxidant compounds may modulate these detrimental effects.

Given the central role of oxidative stress and inflammation in the pathophysiology of insulin resistance and T2DM, pharmacological strategies incorporating antioxidant compounds may help reduce inflammatory burden, restore redox homeostasis, and improve metabolic outcomes. Plant-derived antioxidants, vitamins, and minerals may serve as adjunctive therapies by targeting multiple mechanistic pathways described in this framework, ultimately improving glycemic control and mitigating T2DM-related complications. Although this review evaluates each antioxidant individually, an emerging area of interest is the potential for synergistic or additive interactions between compounds. Antioxidants often target different components of the redox network, such as mitochondrial ROS generation, lipid peroxidation, glutathione cycling, or enzymatic antioxidant pathways, and may therefore complement each other mechanistically. For example, vitamin C can regenerate vitamin E, NAC enhances intracellular glutathione levels, and alpha-lipoic acid can restore multiple antioxidant systems simultaneously, suggesting that combined use could amplify protective effects on glucose homeostasis and oxidative stress. Similarly, polyphenols such as curcumin, quercetin, and resveratrol activate overlapping but not identical signaling pathways such as AMPK, Nrf2, and SIRT1, raising the possibility of multi-target synergy when used together. Evidence for such interactions in T2DM, however, is limited and mostly derived from preclinical studies. A few small human trials have examined combinations, for example, ALA with acetyl-L-carnitine, or GlyNAC, reporting improvements in mitochondrial function, insulin sensitivity, or oxidative stress markers beyond what is typically observed with single agents. Nonetheless, these studies are short in duration, underpowered, and often use heterogeneous formulations, making it difficult to draw firm conclusions. As a result, the clinical relevance of synergistic antioxidant therapy remains largely speculative. Future research should evaluate whether rationally designed combinations, particularly those that address distinct aspects of redox imbalance, offer greater therapeutic benefit than monotherapy. Factorial RCT designs, comparative combination trials, and mechanistic studies using biomarkers of oxidative stress, mitochondrial function, and inflammation will be essential for determining whether synergy exists and how it may translate into meaningful clinical outcomes for individuals with T2DM. Combining antioxidant agents is mechanistically appealing in T2DM because different compounds act at complementary nodes of the redox–inflammation–metabolism network: thiol donors (N-acetylcysteine/GlyNAC) restore intracellular glutathione pools, enzymatic mimetics (SOD/GPx mimetics) clear reactive species, lipophilic antioxidants (vitamin E, CoQ10) protect membranes, and polyphenols (curcumin, resveratrol, quercetin, catechins) modulate signaling pathways (AMPK, SIRT1, Nrf2, NF-κB). Rationally paired agents can therefore provide additive protection or true synergy [237]. Early human and translational data provide proof-of-concept but remain preliminary. For example, GlyNAC (glycine + NAC) improves glutathione redox status and mitochondrial function in short clinical studies, suggesting benefit when combined with mitochondrial cofactors such as alpha-lipoic acid or CoQ10 [237]. Reviews of CoQ10 often highlight enhanced effects when administered with other nutrients such as L-carnitine and arginine although trials are heterogeneous and small [238]. A substantial proportion of the available supportive evidence is derived from preclinical studies or from human trials that are limited by small sample sizes and short follow-up periods. Heterogeneity in formulations and persistent pharmacokinetic challenges, particularly those related to bioavailability, further complicate interpretation of efficacy. Moreover, excessive or improperly targeted antioxidant dosing has the potential to perturb physiological redox signaling or to exhibit pro-oxidant activity. Accordingly, rigorously designed factorial randomized controlled trials employing standardized, bioavailability-validated formulations and incorporating mechanistic biomarkers like GSH:GSSG ratios, assessments of mitochondrial respiration, and indices of Nrf2 pathway activation are required to ascertain whether specific combinations confer clinically meaningful benefit. While observational studies associating higher dietary antioxidant capacity with reduced risk of T2DM provide conceptual support for multi-component approaches, such evidence remains insufficient to substitute for randomized evaluations of defined combination interventions [239].

A major limitation for several antioxidant compounds discussed in this review—particularly quercetin, curcumin, resveratrol, and CoQ10—is their inherently low oral bioavailability. This issue has important clinical implications because many promising mechanistic effects demonstrated in vitro occur at concentrations that are difficult to achieve in humans through conventional supplementation. Poor intestinal absorption, rapid metabolism, and limited systemic stability all contribute to low circulating levels, which may partly explain the inconsistent findings across human trials and the modest metabolic improvements observed compared with preclinical models. In the context of T2DM, where sustained antioxidant and anti-inflammatory activity is likely required to influence glycemic control or insulin signaling, suboptimal bioavailability can blunt therapeutic potential and complicate dose–response relationships in clinical research. To address these limitations, several formulation strategies have emerged. Nanoparticle-based delivery systems, liposomal encapsulation, phytosome complexes, and emulsified preparations have demonstrated significantly enhanced plasma concentrations for compounds like curcumin, resveratrol, and quercetin. CoQ10 bioavailability has been improved through solubilized or ubiquinol formulations that bypass some of the absorption bottlenecks of standard ubiquinone. These technologies are increasingly being incorporated into clinical studies, although long-term safety and comparability across formulations remain areas of active investigation. It is also important to distinguish between dietary sources and supplemental forms. Whole-food sources often provide antioxidants bound within complex matrices, accompanied by fiber, lipids, and synergistic phytochemicals that influence absorption and metabolic effects. In contrast, isolated supplements can deliver higher nominal doses but lack the co-factors that naturally enhance uptake, and their pharmacokinetics can differ substantially. These differences may contribute to the stronger epidemiologic associations observed for antioxidant-rich diets compared with the more variable outcomes reported for supplementation trials. Overall, a clearer understanding of how bioavailability shapes clinical response will be essential for designing effective, evidence-based antioxidant interventions for T2DM. Nevertheless, due to the limited clinical data and the poor pharmacokinetic profiles of many of these compounds, further studies are required, both at the clinical level and in the development of pharmaceutical formulations, with improved pharmacokinetic characteristics, despite their highly promising potential. In conclusion, Table 1 provides a comparative evaluation of the most applied non-enzymatic antioxidant compounds against T2DM, summarizing their efficacy, clinical recommendations, and safety profiles.

## Figures and Tables

**Table 1:** Mechanistic targets, clinical outcomes, evidence-based recommendations, and safety profile for the most applied non-enzymatic antioxidant compounds against obesity-associated T2DM.

Non-Enzymatic Antioxidant Compound	Mechanistic Targets	Clinical Outcomes	Evidence-Based Recommendations	Safety Profile
Vitamin E	Vitamin E inhibits activation of p38 MAPK pathway [77], as well as NF-κB, and STAT-3 signaling [78], and decreases the production of pro-inflammatory eicosanoids [79].	Vitamin E exhibits anti-inflammatory [76], antioxidant [74,75], antidiabetic [80], anti-atherogenic properties [74], and has been shown anti-obesity effects in animal models [81].	RCTs and metanalyses show that vitamin E supplementation produces moderate improvement in OS markers; however, effects on glycemic control and autonomic function remain inconsistent [185,240]. Possible benefits for diabetic retinopathy and nephropathy have been reported, although the evidence in not yet conclusive [241,242].	Safety at high doses (>400 IU/day) is uncertain; may interact with anticoagulants such as warfarin [185].
Vitamin C	Unclear and potentially pleitropic [243].	Vitamin C exhibits antioxidant and antidiabetic effects [86,88], shows hypolipidemic effects in postmenopausal women with T2DM [87] and acts synergistically with vitamin E to enhance antioxidative enzyme activity, including SOD and GSH, in patients with T2DM [86].	Short-term RCTs and meta-analyses indicate that vitamin C supplementation produces moderate reductions in fasting glucose and certain OS markers in individuals with obesity-associated T2DM. However, effects on HbA1c remain inconsistent, likely in part due to the short duration of most of the studies (<6 months) [244,245].	Safe at doses below 2000 mg/day for adults aged 19 and older [246]; high doses may cause gastrointestinal discomfort and may increase the risk of oxalate stone formation in susceptible individuals [244].
N-acetylcysteine	N-acetylcysteine inhibits the NF-κB signaling pathway [99], while activating the PI3K/Akt and JNK2/STAT3 pathways, mechanisms relevant to metabolic regulation in T2DM [97].	N-acetylcysteine exhibits anti-inflammatory and antioxidant properties [89,90], and has demonstrated antidiabetic potential [95]. It scavenges ROS [99] and serves as a precursor for GSH synthesis [89]. Additionally, it enhances the activity of key antioxidant enzymes, including SOD [101].	Preclinical studies and small human trials—including GlyNAC investigations—indicate reductions in OS and suggest potential improvements in insulin sensitivity and mitochondrial function in the context of T2DM. Nevertheless, clinical evidence supporting direct glycemic benefits remains limited and preliminary [238,247].	Safe at usual doses (1200–2400 mg/day) [238,248], though gastrointestinal discomfort may occur; caution is advised in individuals with a history of asthma or bronchospasm [249].
Curcumin	Curcumin suppresses key pro-inflammatory signaling pathways relevant to T2DM pathophysiology, including NF-κB and MAPK [184], ERK/JNK [186], IKK [187,190], and JAK/STAT [184]. It also activates regulatory pathways involved in cellular protection and metabolic homeostasis, particularly the PI3K–Akt–GSK3β pathway [186] and the Keap1/Nrf2 antioxidant pathway [184].	Curcumin exhibits anti-inflammatory [184], antioxidant [184], and metabolic regulatory actions [187,189]. It chelates metal ions and scavenges ROS [184], while enhancing the activity of key antioxidant enzymes, including SOD and catalase [184]. Curcumin promotes glucose uptake and improves insulin sensitivity [186], β-cell function [191], and overall glycemic control [187]. Additionally, it reduces triglycerides, total cholesterol, and LDL cholesterol, while increasing HDL levels [188]. Furthermore, curcumin inhibits glucosidase and aldose reductase [186], and has been associated with reductions in body weight [184,187,189], supporting its relevance in obesity-related T2DM.	Meta-analyses of RCTs indicate that curcumin produces moderate reductions in fasting plasma glucose, HbA1c, and inflammatory markers in individuals with T2DM. However, the magnitude and consistency of these effects vary depending on the dose, formulation, and methodological quality of the included studies [250,251,252,253].	Curcumin is generally well tolerated at typical doses of approximately 1500 mg per day when used over extended periods (several months) [252,253,254]. However, higher doses may cause gastrointestinal discomfort and have the potential to interact with anticoagulant medications [252,253,254].
Resveratrol	Resveratrol exerts multiple mechanistic actions relevant to obesity-associated T2DM. It modulates the composition and function of the gut microbiome [196], and inhibits key metabolic and inflammatory regulators including the NF-κB pathway [198], and the PPARγ [196]. It also suppresses PTP1B, thereby permitting sustained activation of the IRS-1/PI3K/AKT signaling cascade [198]. Additionally, it activates several protective and metabolic pathways, including AMPK/SIRT1, Keap1/Nrf2 and Akt [197,198]. Resveratrol further supports mitochondrial function and biogenesis through the activation of FOXO1 and PGC-1α [198].	Resveratrol exhibits antioxidant and anti-inflammatory properties in experimental models of diabetes [197,198]. It promotes lipolysis, while suppressing lipogenesis, thereby contributing to reductions in adiposity and overall body weight [197]. It also exerts protective anti-apoptotic effects on pancreatic β-cells [198]. Furthermore, it demonstrates potent anti-diabetic actions by ameliorating IR and enhancing glucose uptake and metabolism regulation [198].	Several meta-analyses of RCTs indicate that resveratrol produces modest improvements in glucose homeostasis, blood pressure and -in certain conditions- body weight in individuals with obesity-associated T2DM. However, the variability across studies and the generally small effect sizes do not support its use as a standard therapy [203,255,256,257]. Larger, longer-term, well-designed RCTs are still needed to clarify optimal dosing, duration, and patient subgroups, which might benefit the most.	Human resveratrol supplement doses used in clinical studies vary widely, ranging from approximately 8 mg/day to more than 3000 mg/day, with many trials employing doses between 250 and 1000 mg/day [258]. Resveratrol can modulate CYP enzymes and has shown potential interactions with warfarin in preclinical and some human studies [259].
Catechins	Catechins stimulate the PI3K signaling cascade and activate the Nrf2 pathway [158]. Additionally, they activate the PI3K/Akt and AMPK pathways [159].	Catechins exert multiple beneficial effects on obesity-associated T2DM by possessing potent antioxidant and anti-inflammatory properties [153,157], thereby contributing to mitigation of metabolic dysfunction [153,157]. They enhance glucose homeostasis by promoting the translocation of GLUT4 from intracellular compartments to the cell membrane, facilitating increased glucose uptake [159]. They demonstrate anti-obesity properties through the upregulation of UCP1, which stimulates thermogenesis and energy expenditure [160]. They support cardiovascular health by lowering SBP via increased NO bioavailability and reduced ET-1 production [161] and they improve lipid profiles by decreasing LDL levels [162].	Meta-analyses demonstrate small yet consistent reductions in fasting glucose and HbA1c with green tea or catechin supplementation [260], as well as a lower risk of developing type 2 diabetes mellitus (T2DM) associated with habitual intake [261]. Mechanistic and intermediary-marker studies—encompassing gut microbiota modulation, improvements in lipid profiles, and reductions in waist circumference—provide biologically plausible pathways through which catechins may influence metabolic risk in obesity [262]. Overall, the magnitude of these effects is modest [260], and considerable heterogeneity exists across studies due to differences in dosage, study population, intervention duration, and baseline metabolic status. Furthermore, some analyses report no significant improvements in specific glycemic parameters, such as fasting insulin, among individuals with T2DM [263,264,265].	High dose of EGCG ≥ 800 mg/day extracts have been associated with rare cases of serum transaminases, indicating potential hepatotoxicity at excessive intakes [266]. In addition, the caffeine content of many green tea–derived preparations may pose tolerability issues for sensitive individuals [260].
Quercetin	Quercetin exerts its protective actions against obesity associated T2DM by modulating several key molecular pathways. It activates the KEAP1/Nrf2 [169], IRS-1/PI3K/Akt [163,176], AMPK [176] and HO-1 [180] signaling cascades, while concurrently inhibiting the NF-κΒ [171] and SphK1-S1P [181] pathways.	Quercetin demonstrates significant antioxidant [169] and anti-inflammatory activities [171]. It inhibits lipogenesis and mitigates overall adiposity and the development of NAFLD by suppressing the expression of *ACACA, FASN,* and *SREBP-1c* [172,173]; Quercetin also exhibits antidiabetic properties by reducing intestinal glucose absorption, enhancing insulin secretion and sensitivity, promoting GLUT4 translocation to the cell membrane, increasing cellular glucose uptake and protecting pancreatic β-cells [163]. Additionally, it may ameliorate diabetic retinopathy [180] and attenuate diabetic renal fibrosis [181]. Quercetin has been reported to improve endothelial function by increasing NO bioavailability [182] and may also reduce SBP in postmenopausal women with T2DM [183].	The available clinical evidence consists largely of small randomized controlled trials and pilot studies. Recent meta-analyses and systematic reviews indicate modest improvements in oxidative-stress markers, blood pressure, and select metabolic parameters following quercetin supplementation, although findings remain heterogeneous and dose-dependent [267,268]. Individual trials and early open-label studies in individuals with T2DM similarly report benefits in OS biomarkers, quality-of-life indices, and certain cardiometabolic outcomes; however, these studies are limited by small sample sizes, short durations, and considerable variability in quercetin formulations and dosing regimens [269]. Evidence for glycemic outcomes remains limited [268].	Quercetin is generally well tolerated in clinical studies, including those involving individuals with T2DM, with adverse events typically mild and primarily limited to gastrointestinal complaints such as nausea or dyspepsia [269]. It may interact with certain medications through cytochrome P450–mediated mechanisms [270]. Human intervention trials most commonly employ doses of 500–1000 mg per day for short-to-moderate durations, typically 4–12 weeks [268].
Lycopene	Lycopene exerts its protective effects in obesity-associated T2DM through multiple molecular mechanisms. It suppresses activation of the NF-κB signaling pathway and downregulates genes involved in lipogenesis, thermogenetic and mitochondrial functional, including *Fas*, *ACACA*, *PPARγ*, *PGC1α*, *Prdm16*, *UCPs*, and *Sirt1*. Lycopene also reduces the expression of autophagy-related genes such as *Atg7, Atg14, P62, Lc3,* and *Beclin* [214]. Furthermore, it modulates cell-survival pathways by influencing proteins associated with apoptosis and cellular stress responses, including RAGE, NF-қB, Bax, Bcl-Xl, and Bcl-2 [216]; In addition, lycopene enhances the expression of HO-1 in the kidneys of individuals with T2DM [220].	Lycopene exhibits notable antioxidant [206,217] and anti-inflammatory activities [206], enhancing the expression of key antioxidant enzymes, including SOD, GPx, GR, while reducing markers of OS such as the levels of MDA, and XOD [219]. In experimental models, lycopene has been shown to stimulate lipolysis, improve circulating lipid profiles, limit weight gain, and reduce hepatic fat accumulation [213]. Additionally, it demonstrates antidiabetic properties by lowering blood glucose and insulin concentrations [216,222].	Human clinical evidence for lycopene in obesity-associated T2DM is limited and heterogeneous. Small randomized trials and pilot studies have generally reported improvements in OS biomarkers and some lipid parameters after lycopene supplementation, but effects on clinically relevant glycemic outcomes (fasting glucose, HbA1c, IR) are inconsistent and sparsely reported. Recent systematic reviews reach similar conclusions and highlight the need for larger, longer, well-controlled trials to establish efficacy, optimal dose and formulation, and durability of effects [271,272,273].	Lycopene is considered safe when consumed through the habitual diet, with reported adverse effects uncommon and generally mild; however, long-term safety data remain limited [271]. Human supplementation studies outside the diabetes setting have typically used daily doses of 10–30 mg for several weeks to a few months [274]. Higher short-term doses of approximately 70–75 mg/day have also been evaluated without major adverse effects, though these findings are based on small, short-duration trials and should be interpreted with caution [275,276].

## Data Availability

No new data were created or analyzed in this study.

## Abbreviations

ACACA: acetyl-CoA carboxylase; ACY-1: aminoacylase-1; AGEs: advanced glycation end products; Akt: also known as Protein Kinase B (PKB); AMPK: AMP-activated protein kinase; AP-1: activator protein-1; ATP: adenosine triphosphate; ASCVD: atherosclerotic cardiovascular disease; BAT: brown adipose tissue; CoQ10: Coenzyme Q_10_; COX-2: cyclooxygenase-2; CO_3_•^−^: carbon trioxide radicals; COMTs: catechol-O-methyltransferases; CPT1α: carnitine palmitoyltransferase 1A; CVDs: cardiovascular diseases; DKA: diabetic ketoacidosis; DM: diabetes mellitus; EGCG: epigallocatechin gallate; ERK: extracellular signal-regulated kinase; ET-1: endothelin-1; ETC: electron transport chain; FASN: fatty acid synthase; FBG: fasting plasma glucose; FOXO1: Forkhead box protein O1; GCL: glutamate-cysteine ligase (GCL); GDM: gestational diabetes mellitus; GlyNAC: glycine + NAC; GPx: glutathione peroxidase; GSH: glutathione; GSK3: glycogen synthase kinase 3; HbA1c: glycated hemoglobin; HDL: high-density lipoprotein; HFDs: high-fat diets; HO-1: heme oxygenase-1; H_2_O: water; H_2_O_2_: hydrogen peroxide; HRT: hormone replacement therapy; HSP70: heat shock protein 70; IFN-γ: interferon-gamma; IKK: IκB kinase; IL-6: interleukin-6; iNOS: inducible nitric oxide synthase; IR: insulin resistance; IRS-1: insulin receptor substrate-1; JAK: Janus kinase; LDL: low-density lipoprotein; MAPK: mitogen-activated protein kinase; MetS: metabolic syndrome; MDA: malondialdehyde metallothionein; mTOR/S6K1: ribosomal protein S6 kinase 1; NAC: N-Acetylcysteine; NAFLD: non-alcoholic fatty liver disease; NASH: nonalcoholic steatohepatitis; NF-κB: nuclear transcription factor κB; NO: nitric oxide; •NO_2_: nitrogen dioxide radicals; NOX: NADPH oxidase; O_2_: molecular oxygen; O_2_•^−^: superoxide radicals; •OH: hydroxyl radicals; OS: oxidative stress; oxLDL: oxidized low-density lipoprotein cholesterol; p38 MAP-kinases: p38 mitogen-activated protein kinases; PAD: peripheral artery disease; PAI-1: plasminogen activator inhibitor-1; PDR: proliferative diabetic retinopathy; PGC1α: peroxisome proliferator-activated receptor gamma coactivator 1-alpha; PI3K: phosphoinositide 3-kinase; PKCε: protein kinase C epsilon; Prx: peroxiredoxine; PTEN: phosphatase and tensin homolog; PTP1B: protein tyrosine phosphatase 1B; PUFAs: polyunsaturated fatty acids; RNS: reactive nitrogen species; ROS: reactive oxygen species; SBP: systolic blood pressure; SCs: Schwann cells; SFAs: saturated fatty acids; SLNs: solid lipid nanoparticles; SOCS3: suppressor of cytokine signaling 3; SOD: superoxide dismutase; STAT: signal transducer and activator of transcription; STAT-3: signal transducer and activator of transcription 3; STZ: streptozotocin; SULTs: sulfotransferases; TAC: total antioxidant capacity; TC: total cholesterol; T2DM: type 2 diabetes mellitus; TMAO: trimethylamine-N-oxide; UCP2: uncoupling protein 2; UGTs: UDP-glucuronyltransferases; vLDLs: very-low-density lipoproteins; XOD: xanthine oxidase.
